# Supplementary material for: A robust method of nuclei isolation for single-cell RNA sequencing of solid tissues from the plant genus Populus
Source: PLoS One. 2021 May 11;16(5):e0251149. doi: 10.1371/journal.pone.0251149 (PMC8112699; doi:10.1371/journal.pone.0251149)
Supplement: S1 File — (DOCX) [file pone.0251149.s001.docx]

**S1 File.**

**Nuclei isolation protocol for single-nuclei RNA-seq of solid tissues from the plant genus *Populus.***

*All experimental procedures are carried out at 4°C, in a walk-in cold chamber, to minimize mRNA degradation. Similarly, reagents, supplies, and instruments used in the nuclei isolation are cooled at least 30 minutes before use.*

1. Place the plant material on a glass plate with 200-400 µl of the Nuclei Isolation Buffer (NIB, 10 mM MES-KOH pH 5.7, 10 mM NaCl, 10 mM KCl, 3 mM MgCl_2_, 2.5 mM EDTA, 250 mM sucrose, 0.1 mM spermine, 0.5 mM spermidine, 1 mM DTT, 0.5 U/μl Protector RNase Inhibitor). In the current example, 20 dissected shoot apices or one single stem internode were used. The amount of NIB buffer varied depending on the tissue source (200 µl for shoot apices and 400 µl for stem).
2. Chop the sample with a sterile razor blade for 2 minutes. Repeat the procedure with a 30 second interval in between. *Note:* This step may be repeated more than twice, depending on the properties of the tissue.
3. Wash the homogenate with 5 ml of NIB with lower Protector RNase Inhibitor concentration (NIB, 10 mM MES-KOH pH 5.7, 10 mM NaCl, 10 mM KCl, 3 mM MgCl_2_, 2.5 mM EDTA, 250 mM sucrose, 0.1 mM spermine, 0.5 mM spermidine, 1 mM DTT, 0.2 U/μl Protector RNase Inhibitor) from the glass plate into a 50 ml conical tube, using a 2 ml Pasteur pipette.
4. Incubate it on a rocking shaker for 5 minutes, with gentle horizontal shaking.
5. Filter the sample through one layer of miracloth (CALBIOCHEM) pre-wetted with NIB buffer. The miracloth should be laid on top of a 50 ml conical tube placed on ice and tilted on its side. Wash the filter with 1-2 ml of NIB, to increase the nuclei recovered from the miracloth filter membrane.
6. Filter the sample through a 40 μm strainer (Greiner Bio-One) pre-wetted with NIB buffer, laid on top of a 50 ml conical tube placed on ice, tilted on its side. Wash the filter with an additional 1-2 ml of NIB after filtering.
7. Centrifuge the sample at 600 g for 5 minutes at 4°C.
8. After centrifugation, remove the supernatant, and resuspend the pellet in 4 ml of NIB WASH (10 mM MES-KOH pH 5.7, 10 mM NaCl, 10 mM KCl, 3 mM MgCl2, 250 mM sucrose, 0.1% BSA and 0.2 U/μl Protector RNase Inhibitor) pipetting very gently with a 2 ml Pasteur pipette.
9. Centrifuge the sample at 600 g for 5 minutes at 4°C.
10. Repeat the step 8 for a total of two wash steps. After the second wash, resuspend the pellet is 750-1000 μL of NIB WASH.
11. Stain the nuclei by adding 5 μg/mL DAPI, for 5 minutes at room temperature.
12. Transfer the sample to a cell sorter, to obtain a minimum of 40,000 nuclei at a final concentration of approximately 500 nuclei/µl. Collect the nuclei into 1.5 ml RNase free non-stick Eppendorf low binding tubes (Thermofisher) containing 10μl of NIB WASH. ***First quality checkpoint: check the overall nuclei integrity under a confocal microscope before and after sorting (Fig. S1A).***
13. Load 20,000 nuclei into the 10× Genomics microfluidic chip, following the Single Cell v3.1 Dual Index Gene Expression protocol. NIB WASH is added to the 40 μl (500 nuclei/ μl) of nuclei suspension instead of water to reach the final volume required (43.2 μl). Then, add the 31.8 μl of the cDNA master mix slowly to the nuclei suspension to minimize the osmotic shock. Perform cDNA amplification according to the Single Cell v3.1 Dual Index Gene Expression protocol, for 15 cycles. ***Second quality checkpoint: confirm the cDNA quality by observing its profile in the Agilent D5000 High Sensitivity Tape Station (Fig. S1B).***
14. Finally, construct the snRNA-seq Dual Index library following the Single Cell v3.1 Dual Index Gene Expression protocol. ***Third quality checkpoint: Evaluate the library profile using the Agilent Tape Station to confirm the high quality of the library (Fig. S1C).***
