# Supplementary figures and images for: A robust method of nuclei isolation for single-cell RNA sequencing of solid tissues from the plant genus Populus
Source: PLoS One. 2021 May 11;16(5):e0251149. doi: 10.1371/journal.pone.0251149 (PMC8112699; doi:10.1371/journal.pone.0251149)

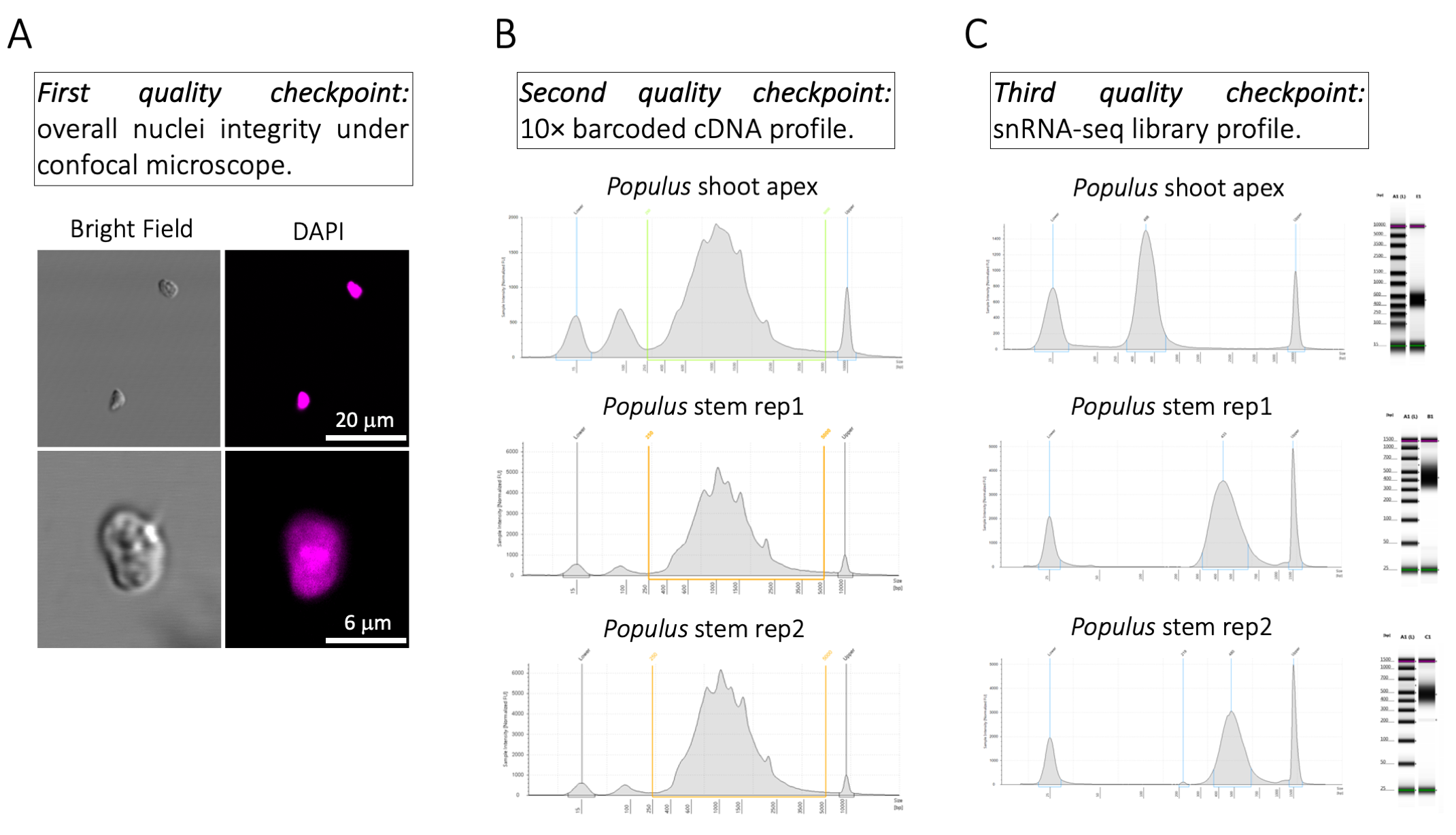

Supplement: S1 Fig — (A) nuclei integrity is examined under the scope before and after nuclei sorting by FANS. After FANS, we also evaluate the absence of debris and organelles to determine the FANS’ effectiveness. (B) cDNA yield and quality can only be determined after the cDNA amplification step. The good quality of the cDNA from Populus shoots and stem was confirmed by observing their profile in the Agilent Tape Station. (C) The final snRNA-seq libraries are also checked in the Agilent Tape Station to evaluate their quality. (TIF) [file pone.0251149.s002.tif]

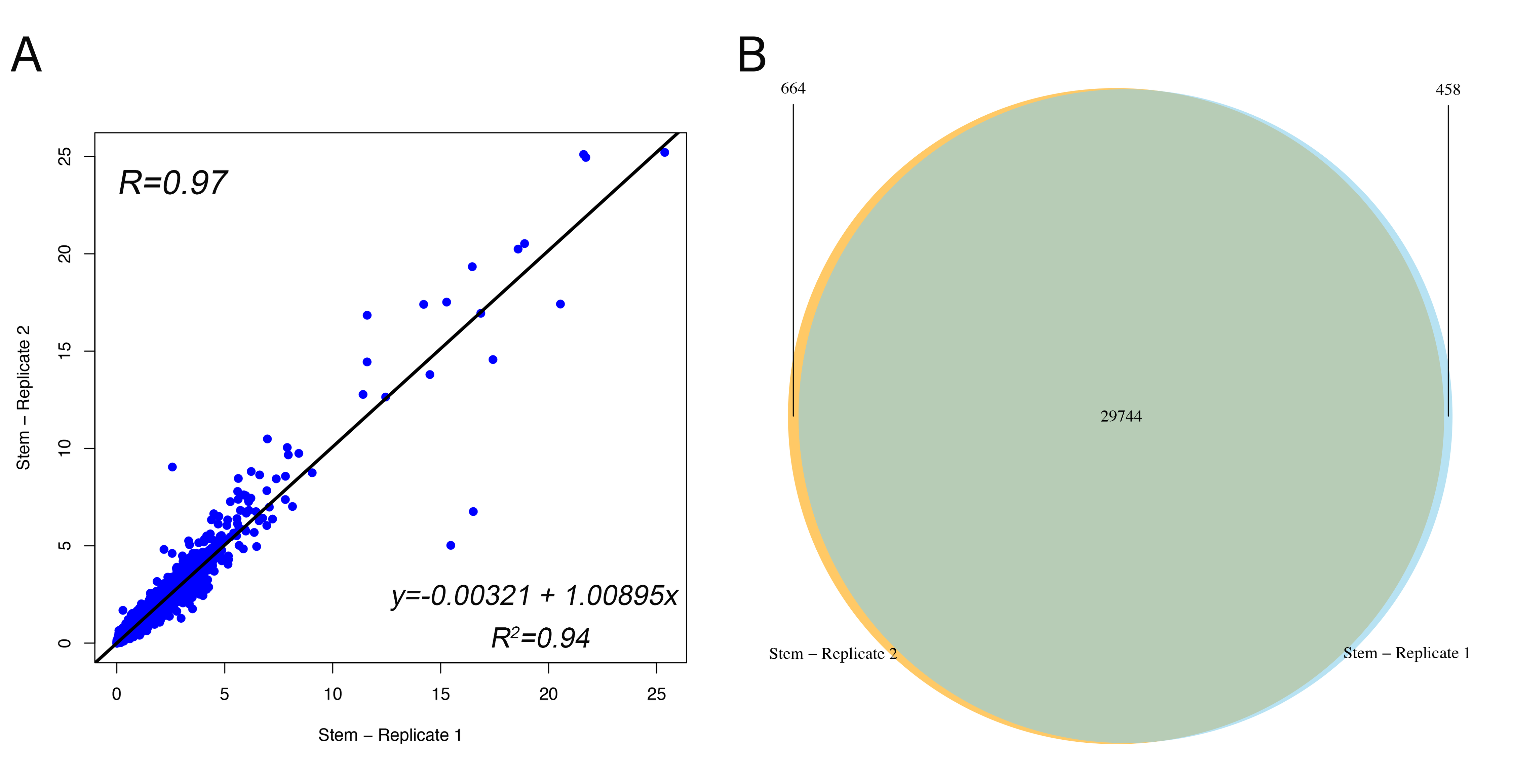

Supplement: S2 Fig — (A) Correlation of the normalized gene expression between the two biological replicates performed using Populus stem samples. The high correlation between both biological replicates (R = 0.97) suggests a good reproducibility of the snRNA-seq when following the protocol developed in the present research work. (B) Venn diagram showing the commonality in the expressed genes between Populus stem snRNA-seq replicate 1 and replicate 2 data. (TIF) [file pone.0251149.s003.tif]
