# Supplementary material for: A robust method of nuclei isolation for single-cell RNA sequencing of solid tissues from the plant genus Populus
Source: PLoS One. 2021 May 11;16(5):e0251149. doi: 10.1371/journal.pone.0251149 (PMC8112699; doi:10.1371/journal.pone.0251149)
Supplement: Attachment — Submitted filename: Comments.pdf [file pone.0251149.s004.pdf]

General comments-

Conde et al. has proposed an extended method for nuclei preparation from complex plant samples for single cell sequencing analysis which is key challenge for plant researchers. The introduction is very concise and well composed and I must appreciate it. The optimized protocol is very nicely descried in method sections that supports its possible application for single cell RNA-Seq analysis in other complex plants. Results and discussion are well written in context to key precautions and challenges associated with nuclei isolation and downstream single cell sequencing. The extended methodology proposed in this research paper will facilitate further developments of scRNA-Seq analysis in many other important plant systems. Overall, the current manuscript satisfies all the necessary points to be published in Plos ONE, hence I recommend it for publication in this journal.

Some minor corrections/ comments

Comment 1: Correct “can be only be” to “can only be” in S1 file line 76.
